# Differential Peripheral Blood Gene Expression Profile Based on Her2 Expression on Primary Tumors of Breast Cancer Patients

**DOI:** 10.1371/journal.pone.0102764

**Published:** 2014-07-28

**Authors:** Oana Tudoran, Oana Virtic, Loredana Balacescu, Laura Pop, Flaviu Dragla, Alexandru Eniu, Bogdan Fetica, Ovidiu Balacescu, Ioana Berindan-Neagoe

**Affiliations:** 1 Department of Functional Genomics and Experimental Pathology, The Oncology Institute I. Chiricuta, Cluj-Napoca, Romania; 2 Research Center for Functional Genomics, Biomedicine and Translational Medicine, Iuliu Hatieganu University of Medicine and Pharmacy, Cluj-Napoca, Romania; 3 Department of Pathology, The Oncology Institute I. Chiricuta, Cluj-Napoca, Romania; 4 Department of Immunology, Iuliu Hatieganu University of Medicine and Pharmacy, Cluj Napoca, Romania; University of Navarra, Spain

## Abstract

Breast cancer prognosis and treatment is highly dependent on the molecular features of the primary tumors. These tumors release specific molecules into the environment that trigger characteristic responses into the circulatory cells. In this study we investigated the expression pattern of 84 genes known to be involved in breast cancer signaling in the peripheral blood of breast cancer patients with ER-, PR- primary tumors. The patients were grouped according to Her2 expression on the primary tumors in Her2+ and Her2- cohorts. Transcriptional analysis revealed 15 genes to be differentially expressed between the two groups highlighting that Her2 signaling in primary tumors could be associated with specific blood gene expression. We found CCNA1 to be up-regulated, while ERBB2, RASSF1, CDH1, MKI67, GATA3, GLI1, SFN, PTGS2, JUN, NOTCH1, CTNNB1, KRT8, SRC, and HIC1 genes were down-regulated in the blood of triple negative breast cancer patients compared to Her2+ cohort. IPA network analysis predicts that the identified genes are interconnected and regulate each other. These genes code for cell cycle regulators, cell adhesion molecules, transcription factors or signal transducers that modulate immune signaling, several genes being also associated with cancer progression and treatment response. These results indicate an altered immune signaling in the peripheral blood of triple negative breast cancer patients. The involvement of the immune system is necessary in favorable treatment response, therefore these results could explain the low response rates observed for triple negative breast cancer patients.

## Introduction

Breast cancer has the highest incidence of all cancers among women worldwide, being the leading cause of cancer death in female population (World Health Organization, 2013). Breast cancer, as all solid malignancies, is a heterogeneous disease, patients prognosis being highly dependent on the epidemiological, phenotypic and tumor molecular features [1]–[8]. Molecular characterization of primary tumors has guided breast cancer management towards personalized treatment facilitating the selection of specific adjuvant therapy for those who are most likely to benefit. Currently, breast cancers are classified and treated based on estrogen receptors (ER), progesterone receptors (PR), epidermal growth factor receptor 2 (Her2) expressions, and proliferation, usually assessed by Ki-67 expression. Around 78% of breast cancers are luminal tumors, characterized by the presence of ER, PR, the rest of 22% being nonluminal tumors negative for ER, PR expression [1], [2], [8]. These two subtypes can be further divided into Her2 overexpressing and Her2 negative tumors. A 15 years retrospective survival study [8] showed that patients with nonluminal tumors have the worst 5 years overall and relapse free survivals rates. However, for the Her2+ nonluminal patients, promising outcome improvement have been achieved after Trastuzumab approval as adjuvant therapy (rev in [9]) in 2006. Currently, the worst prognosis have the triple negative tumors (ER-, PR-, Her2-) which account for 10–20% of all breast cancers [8], [10], and are characterized by a more aggressive behavior and increased risk of metastases. Due to lack of well-characterized molecular targets, hence therapeutics, chemotherapy is the only available systemic treatment option for these tumors. Therefore, efforts have been focused on characterizing and finding new targeting therapeutic approaches, but also predictive prognostic molecules in order to avoid patient over or under treatment.

Clinical outcome of a patient is determined only in part by the primary tumor; tumor cells do not manifest the disease alone, but rather corrupt the whole body. Previous studies have showed that cancer cells [11]–[13], including breast cancer cells [14], [15] induce specific changes in the blood environment, triggering characteristic responses in blood cells. Blood samples are minimal invasive investigation specimens, but also represent the physiological state of the body, making blood sampling an attractive alternative to the invasive tumor sampling.

In line with this view, in this pilot study, we investigated the expression profile of 84 genes involved in breast cancer in the peripheral blood samples collected from TNBC patients and compared them to those from ER-, PR-, Her2+ patients. Our results indicate that the two groups show distinct expression patterns in the peripheral blood.

## Materials and Methods

### Ethics Statement

The Oncology Institute I. Chiricuta Ethics Committee approved the study and all participant patients gave their written informed consent.

### Patients and blood samples

In this study were enrolled 30 breast cancer patients that were diagnosed at The Oncology Institute Ion Chiricuta Cluj-Napoca, Romania between 2010 and 2012. The patients were enrolled consequently as they presented for diagnosis. The institutional research ethics committee approved the study and all participant patients gave their written informed consent. Histopathology analysis and staging of the patients was done according to the AJCC criteria. Estrogen, progesterone and Her2 receptors status were analyzed by immunohistochemistry, Her2 gene amplification was tested by CISH when suitable. Only patients with ER -, PR - were further considered for this study.

Blood samples were collected before the patients underwent any treatment, in a four hours interval (8–12 am) on EDTA anticoagulant tubes. After plasma and red blood cells removal the nucleated cells were processed for RNA isolation according to the classical protocol using TriReagent and further purified with RNeasy Mini Kit (Qiagen, Romania). Al the RNA samples had RIN (RNA Integrity Number) greater than 7. Additionally, the complete blood count was performed for each patient. The patients' clinicopathological characteristics are presented in Table 1.

**Table 1:** Clinicopathological features of patients.

No	Age	HER2 status	Clinical stage	TNM	Nottingham grading	Menopause age
1	35	−	II B	T2N1M0	III	−
2	58	−	III B	T4bN3M0	II	50
3	58	−	II B	T2N1M0	II	−
4	59	−	III A	T3N2M0	III	N/A
5	45	−	III A	T3N1M0	III	−
6	48	−	II B	T2N1M0 (r*), T1N0M0 (l*)	III	32
7	51	−	III B	T4bN2M0	I	−
8	49	−	II B	T2N1M0	III	−
9	50	−	III B	T4bN1M0	II	−
10	55	−	III B	T4bN2M0 (r*), T1N0M0 (l*)	III	51
11	59	−	III B	T4cN2M0	III	52
12	42	−	N/A	T4bN2Mx	III	38
13	40	−	II B	T2N1Mx	III	−
14	74	−	III B	T4bN2M0	III	48
15	56	−	II B	T2N1M0	III	N/A
16	56	−	III B	T4bN1M0	III	46
17	59	−	I	T1cN0Mx	I	49
18	60	−	II A	T1N1MO	III	45
19	62	+	III C	T2N3M0	III	N/A
20&	42	+	III B	T4bN2M0	I	−
21	42	+	III B	T4bN2Mx	III	−
22	55	+	III B	T2N2aM0	III	54
23	66	+	III B	T4bN2M0	III	53
24	53	+	III A	T2N2M0	III	51
25	61	+	III B	T4bN2M0	III	48
26	44	+	III B	T4bN1M0	III	N/A
27	64	+	III B	T4bN2M0	III	55
28&	83	+	III C	T4dN3M0	III	N/A
29	56	+	N/A	N/A	III	50
30	57	+	III A	T3N2M0	II	53

& Invasive lobular carcinoma.

*Patients with bilateral breast cancer r – right breast tumor; l- left breast tumor.

### PCR array

300 ng of total RNA was reverse transcribed with RT2 First Strand kit, diluted and amplified in 96 well Human Breast Cancer PCR Array plates PAHS-131Z (Qiagen, Romania). The array consists of 84 primer sets for genes involved in breast cancer (Table 2), 5 housekeeping genes for sample-to-sample normalization, and several controls for reverse transcription and PCR reactions. Cycling program settings were done as instructed by the manufacturer and maintained with LightCycler 480 II apparatus (Roche, Romania). SYBR green was used for real time detection and threshold cycles (Ct) were calculated using automated second derivative analysis method.

**Table 2:** Functional grouping of the 84 genes analyzed in the Human Breast Cancer PCR Array.

**Tumor Classification Markers:**
Luminal A–C: FOXA1, TFF3, GATA3, ESR1, KRT8, KRT18, SLC39A6, XBP1, HER2-like: ERBB2, GRB7.
Basal-like/Triple Negative: EGFR, BIRC5, KRT5, NOTCH1.
Metastasis to Lung: PTGS2, ID1, MMP2.
**Transcription Factors:** AR, ESR1, ESR2, FOXA1, CTNNB1, GATA3, HIC1, JUN, MYC, TP53, TP73, NOTCH1, NR3C1, PGR, PRDM2, RARB, RB1, XBP1.
**Signal Transduction:**
Steroid Receptor-Mediated: AR, BRCA1, PGR, CCNE1, ESR1, ESR2, IGF1, KRT19, CTNNB1, RB1.
Hedgehog: CCND1, BCL2, GLI1, SNAI2.
Glucocorticoid: NME1, IGFBP3, NR3C1.
WNT: CTNNB1, APC, CCND1, SFRP1.
PI3K/AKT: AKT1, IGF1, ERBB2, IGF1R, PTEN.
NOTCH: NOTCH1, BIRC5.
MAPK: TP73, MAPK1, MAPK3, MAPK8.
**Angiogenesis:** VEGFA, CTNNB1, CDH13, EGF, ID1, IL6, ERBB2, JUN, SERPINE1, NOTCH1, PLAU, PTEN, SLIT2, THBS1.
**Adhesion:** CDH1, CDH13, ADAM23, APC, BCL2, PTEN, CDKN2A, CSF1, CTNNB1, EGFR, TGFB1, THBS1, ERBB2.
**Proteolysis:** MMP2, MMP9, ADAM23, CST6, CTSD, PLAU, PYCARD.
**Epithelial to Mesenchymal Transition:** NOTCH1, SRC, CTNNB1, TGFB1, TWIST1.
**Cell Cycle:** CCNA1, CCND1, APC, BCL2, CCND2, MYC, PTEN, RASSF1, CCNE1, CDK2, CDKN1A, CDKN1C, CDKN2A, JUN, MKI67, RB1, SFN, TP53.
**DNA Damage:** TP53, TP73, APC, ATM, CCND1, CDKN1A, MAPK1, BRCA1, BRCA2, MGMT, MLH1, SFN.
**Apoptosis:** TP53, TP73, AKT1, BAD, BCL2, CDH1, CDKN1A, CDKN2A, GSTP1, IGF1, IL6, JUN, MUC1, NME1, RARB, APC, SFN, SFRP1, TWIST1.
**Xenobiotic Transport:** ABCB1, ABCG2.

### Data analysis

Correlations between clinical data were analyzed using Fischer exact test or chi square test. The PCR array results were analyzed using ΔΔC_t_ based fold-change calculations with Ct cut-off at 35 cycles. We considered of interest genes with −1.5< fold change >1.5 and an adjusted p value <0.05 according to Benjamini and Hochberg method. The patients were grouped according to Her2 expression on primary tumor at diagnosis. List of differentially expressed genes with associated fold regulation values was uploaded into Ingenuity Pathway Analysis (IPA) software (Ingenuity Systems) for functional analysis. Using information stored in the Ingenuity Knowledge Base (IKB), genes were mapped to networks and biological functions. The significance of the association between genes/networks and biological functions was evaluated by right-tailed Fischer's exact test (p<0.05).

## Results

### Her2 expression and clinicopathological characteristics

Her2 receptor was expressed in the primary tumors of 18 patients, while the rest had either no protein expression or gene amplification in CISH. 33% of the patients were under 50 years at the time of diagnosis with a mean age of 43.4 years, while the over 50 years patients had a mean age of 60 years. Half of all the patients had reached menopause at the time of diagnosis. Twenty-eight patients had invasive ductal carcinomas, and two were diagnosed with invasive lobular carcinoma. Approximately 75% of the patients had tumors in stage III at the time of diagnosis. Almost all patients had positive lymph nodes, but with no detectable secondary distant tumors. Individual clinicopathological features are presented in Table 1. All the patients presented nucleated cell counts within the reference values, without significant differences between the two groups (Table 3).

**Table 3:** Patients' differential blood count according to Her2 expression on the primary tumors.

Cell type	Reference values	Her2-	Her2+	p value
White blood cells	4000–10000/Ul	7287	7239	0.94
Neutrophils	2000–8000/uL	4867	4755	0.83
	45–80%	66	65	0.5
Eosinophils	50–700/uL	88	143	0.17
	= <1,5%	1	2	0.17
Basophils	= <200/uL	22	28	0.21
	= <0,2%	0.29	0.41	0.17
Lymphocytes	1000–4000/uL	1761	1768	0.96
	20–55%	25	25	0.79
Monocytes	300–1000/uL	543	545	0.97
	= <15%	8	8	0.69

Correlations between the clinical data were done in respect to Her2 expression in primary tumors (Table 4). No association was observed for age, clinical stage, tumor size and grading, or menopausal status. Statistical significance was observed between the lymph node status and Her2 expression (p<0.03), less Her2 positive patients having operable mobile ipsilateral axillary lymph nodes than Her2 negative patients.

**Table 4:** Her2 expression and clinicopathological characteristics.

Patient Characteristics	No of patients	%	Her2+	Her2-	P value
**All Patients**	30	100	12	18	
**Age**					
≤50	10	33.33	3	7	0.69
>50	20	66.66	9	11	
**Clinical stage**					
1	1	3.33	0	1	-
2	7	23.33	0	7	
3	20	66.66	11	9	
**Tumor size**					
T1 and T2	11	36.66	3	8	0.44
T3 and T4	18	60	8	10	
**Lymph Nodes**					
N0	1	3.33	0	1	
N1	11	36.66	1	10	0.03
N2	14	46.66	8	6	
N3	3	10	2	1	
**Nottingham grading**					
I	3	10	1	2	0.76
II	4	13.33	1	3	
III	23	76.66	10	13	
**Menopausal status**					
Pre	9	30	2	7	0.4
Post	16	53.33	7	9	

Percentage <100% are attributed to missing information.

### Her2 expression and blood gene profile

**Figure 1:**
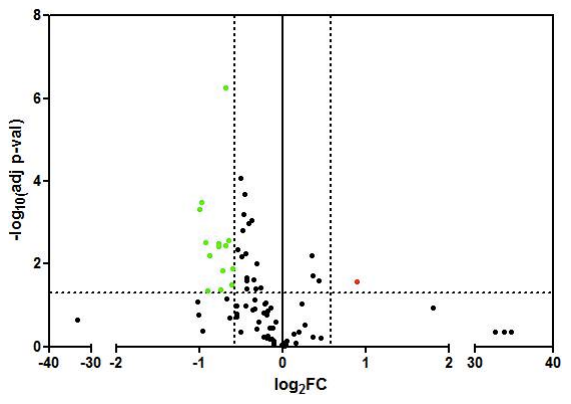
Vulcano plot comparing the 84 gene expression between the Her2+ and TNBC patinets. The genes are represented as log2fold change based on -log10(adjp-value). According to the cut-off of 1.5 fold change (FC) and adjusted p-value of 0.05, the genes in green are underexpressed and the gene in red is overexpressed in TNBC patients compared to Her2+ patients.

We profiled the expression of 84 genes involved in regulation of signal transduction and biological pathways of breast carcinogenesis, progression and invasion. The array contains genes used for tumor classification, signaling molecules and signaling pathways like cell cycle, apoptosis, adhesion and angiogenesis (Table 2). The graphical representation of the genes fold change distribution based on p-value is presented in Figure 1. Transcriptional analysis identified a group of 15 genes to be differentially expressed between Her2- and Her2+ patients (Table 5). With the exception of Cyclin A1, which was upregulated, all the other genes were downregulated in the blood of Her2- patients. These genes are coding cell cycle regulators: *CCNA1*, *JUN*, *MKI67*, *RASSF1*, *SFN*; molecules involved in cell adhesion: *CDH1*, *CTNNB1*, *HER2*, transcription factors: *CTNNB1*, *GATA3*, *HIC1*, *JUN*, *NOTCH1*; or signal transducers: *GLI1*.

**Table 5:** Gene differentially expressed between the Her2- and Her2+ patients.

Symbol	Description	Fold Regulation	Adjusted p value
CCNA1	Cyclin A1	1.9	0.03
ERBB2	V-erb-b2 erythroblastic leukemia viral oncogene homolog 2	−2.0	0.00
RASSF1	Ras association domain family member 1	−2.0	0.00
CDH1	Cadherin 1, type 1, E-cadherin	−1.9	0.05
MKI67	Antigen identified by monoclonal antibody Ki-67	−1.9	0.00
GATA3	GATA binding protein 3	−1.8	0.01
GLI1	GLI family zinc finger 1	−1.7	0.00
SFN	Stratifin	−1.7	0.00
PTGS2	Prostaglandin-endoperoxide synthase 2	−1.7	0.04
JUN	Jun proto-oncogene	−1.6	0.01
NOTCH1	Notch 1	−1.6	0.00
CTNNB1	Catenin (cadherin-associated protein), beta 1	−1.6	0.00
KRT8	Keratin 8	−1.6	0.00
SRC	V-src sarcoma (Schmidt-Ruppin A-2) viral oncogene homolog	−1.5	0.03
HIC1	Hypermethylated in cancer 1	−1.5	0.01

**Figure 2:**
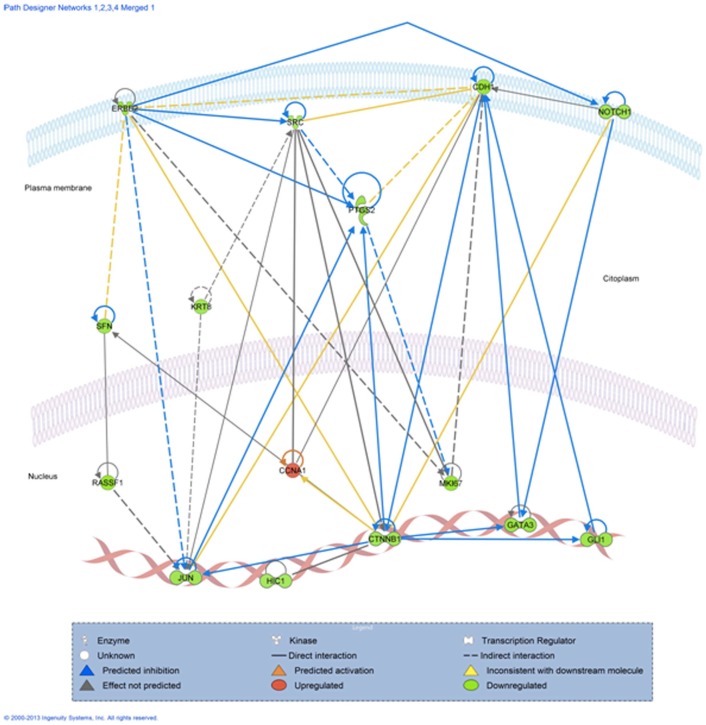
IPA prediction of the interactive network between the differentially expressed genes in the blood of triple negative compared to Her2+ breast cancer patients.

We further used IPA software to understand the chemical, molecular and cellular interaction between these genes in the context of cellular phenotype and disease. Based on gene expression, the software predicted that cell cycle (p = 9.21E-15-3.99E-04) is the top biological function mediated by these genes, followed by cellular movement (p = 4.31E-13-6.01E-04) and development (p = 2.40E-11-5.69E-04). As expected, all these genes were included in the molecular signaling in cancer canonical pathway, specifically in the reproductive system (p = 1.68E-13-4.22E-04), but also can be involved in the hematological system development and function (p = 1.18E-05-5.17E-04). The software also generated 3 possible regulatory networks, which we merged in order to get a more comprehensive view on how these genes are regulated and they might interact with each other (Figure 2).

## Discussion

It is well known that there is an active crosstalk between the tumor, surrounding stromal and adjacent tissues, as well as the immune system. Tumors release a broad range of signaling molecules into the bloodstream which induce changes in the blood cells that can be associated with distinct molecular blood signatures [14]. As breast cancer molecular subtypes differ in terms of treatment and survival, in this pilot study we were focused on investigating whether ER-, PR- patients present specific blood molecular features based on Her2 expression on primary tumors. Our results show that 15 genes are differently regulated in the peripheral blood of these patients, highlighting that Her2 signaling in primary tumors can be associated with specific blood gene expression. Several groups have previously showed a significant relationship between molecular subtypes and the axillary lymph nodes in breast cancer patients [16], [17]. We also associated Her2 signaling with the clinical nodal status, emphasizing a very complex regulatory network between Her2 signaling in the primary tumor, the nodal status and blood microenvironment.

Increasing evidence suggests that breast tumors shed tumor cells into the bloodstream as circulating tumor cells, and one could argue that the differences in expression we observed might come from these cells. A recent study showed that around 24% of the breast cancer patients in stage I-III non-metastatic present circulating tumor cells (CTCs) [18]. We did not measure the number of CTCs in our samples at the time of collection; however, the PCR array plates include detection of cytokeratin-19 (CK-19), which is being used as a marker for detecting CTCs [19], [20]. The amplification cycles for CK-19 in our samples were over the cut-off value (Ct>35 cycles) thus we considered our samples negative or low for CTCs.

Our data suggest that not only tumor cells are characterized by a Her2 dependent signaling pathway, but also the white blood cells [21], [22]. We observed statistically lower Her2 mRNA blood levels for Her2- patients when compared to Her2+ group, in accordance to previous studies which showed that blood Her2 expression correlates with Her2 levels in tumor cells [23], [24]. Network analysis (Figure 2) revealed that the 15 genes are interconnected and regulate each other. Signaling seems to be transduced through several membrane bound receptors, Her2 has been showed to co-localize, interact [25] and regulate Notch1 [26] and SRC [27] expression. Notch1 also regulates CDH1 expression by changing DNA methylation levels of its promoter [28]. These transmembrane proteins further transmit the signal throughout cytoplasmic molecules (SFN, KRT8, SRC or PTGS2) [27], [29] or directly to transcription factors (JUN, CTNNB1, GATA3, GLI1, HIC1) [30] in the nucleus.

The main function mediated by these genes seems to be the cell cycle. However, whether cell cycle is promoted or inhibited it is not clear, as some of these genes are known to increase cell cycle progression, whereas some inhibit cell cycle [31]. Most of these genes have been previously described to be involved in the regulation of immune cells proliferation, differentiation and activation. Her2 [22] and SRC [32] induce myeloid cells proliferation, NOTCH1 [33] and GATA3 [34] regulate thymocytes and T lymphocytes, SFN [35] and PTGS2 [36] signal differentiation of B cells, etc. We found reduced mRNA levels for these genes in TNBC patient's blood, therefore, based on IPA prediction, our hypothesis is that these patients might present an altered immune phenotype compared to Her2+ patients. We did not observe significant differences in the nucleated cell counts between the two groups (Table 3), therefore to support this, immunophenotyping studies could have been carried, but we did not consider this at the time of blood collection.

Studies have shown that immune system modulates treatment outcome in breast cancer patients [37] and of all, the worst responding are those with TNBC tumors [8]. Higher tumor-infiltrating lymphocytes (TILs) have been associated with increased likelihood of pathological response [38], [39] therefore supporting the immune surveillance theory. The molecular mechanism that leads to lymphocyte infiltration is still not completely understood. However, TILs assessment at baseline in TNBC and Her2+ patients could separate high and low risk population [37], moreover it has been showed that some therapies could lead to increased immune reactions. Trastuzumab mediate in part its effect through directing macrophages and natural killer cells towards tumor site [40] as a consequence of innate immunity activation. There is a common believe that conventional therapies could work only in patients that have immune systems that are either pre-activated or that become activated upon treatment, therefore one future development is to find drugs that can reverse immune defects.

On the other hand, part of the identified genes has been previously associated with tumor progression, prognosis and treatment monitoring. Elevated levels of Cyclin A1 are needed to promote tumorigenic behavior in various solid tumors [41]–[43], including breast cancer [44]. A previous study showed that this gene is hypermethylated in white blood cells from cervical cancer patients, and has been associated with a more invasive phenotype [45]. In acute myeloid leukemia patients, high expression of Cyclin A1 mRNA was associated with increased survival [46].

Her2 levels have been proposed for disease monitoring during patient follow up [23]. Early stage breast cancer patients with detectable levels of Her2 in the blood had lower disease free survival and overall survival than Her2 negative patients and Her2 detection was independently associated with early relapse [47]. However, in metastatic breast cancer patients, high blood HER2 mRNA levels were marginally associated with longer overall survival [48].

RASSF1, CDH1 and HIC1 are frequently silenced due to hypermethylation in breast cancer patients [49]–[54]. Several studies previously showed that peripheral blood contains epigenetic information [49], [51], we found RASSF1, CDH1 and HIC1 genes to be significantly underexpressed in the blood cells from Her2 negative patients, levels that could be explained if these patients exhibited higher degrees of promoter methylation than Her2 positive patients. These genes can be hypermethylated not only in breast tumor cells, but also in blood cells [55], [56]. RASSF1 has been previously associated with Her2 status [54] and promoter hypermethylation has been showed to have prognostic values in early stage breast cancer [52], [53].

Overall, our data show that Her2 expression on primary tumors induce differential expression pattern in the peripheral blood of breast cancer patients which can be associated with altered immune signaling pathways. These results could explain the discrepancies in treatment response of Her2+ compared to triple negative breast cancer patients suggesting that baseline monitoring of the immune status may aid in the prediction of treatment response.
