# Correction: Differential Peripheral Blood Gene Expression Profile Based on Her2 Expression on Primary Tumors of Breast Cancer Patients

**DOI:** 10.1371/journal.pone.0110962

**Published:** 2014-10-13

**Authors:** The PLOS ONE Staff

The second affiliation for the third author is not indicated. Loredana Balacescu is affiliated with: 1 Department of Functional Genomics and Experimental Pathology, The Oncology Institute I. Chiricuta, Cluj-Napoca, Romania, and 2 Research Center for Functional Genomics, Biomedicine and Translational Medicine, Iuliu Hatieganu University of Medicine and Pharmacy, Cluj-Napoca, Romania.

## References

[1] Tudoran O, Virtic O, Balacescu L, Pop L, Dragla F, et al (2014) Differential Peripheral Blood Gene Expression Profile Based on Her2 Expression on Primary Tumors of Breast Cancer Patients. PLoS ONE 9(7): e102764 doi:10.1371/journal.pone.0102764 25068292 10.1371/journal.pone.0102764 PMC4113305

